# Anxiety, depressive symptoms, and distress over the course of the war in Ukraine in three federal states in Germany

**DOI:** 10.3389/fpsyt.2023.1167615

**Published:** 2023-04-27

**Authors:** Janka Massag, Sophie Diexer, Bianca Klee, Daniela Costa, Cornelia Gottschick, Anja Broda, Oliver Purschke, Nils Opel, Mascha Binder, Daniel Sedding, Thomas Frese, Matthias Girndt, Jessica Hoell, Irene Moor, Jonas Rosendahl, Michael Gekle, Rafael Mikolajczyk

**Affiliations:** ^1^Institute for Medical Epidemiology, Biometry and Informatics (IMEBI), Interdisciplinary Centre for Health Sciences, Medical Faculty of the Martin Luther University Halle-Wittenberg, Halle (Saale), Germany; ^2^Department for Psychiatry and Psychotherapy, University Hospital, Friedrich Schiller University Jena, Jena, Germany; ^3^German Center for Mental Health, Site Jena-Magdeburg-Halle, Jena, Germany; ^4^Department of Internal Medicine IV, Oncology/Haematology, Martin Luther University Halle-Wittenberg, Halle (Saale), Germany; ^5^Department of Cardiology and Intensive Care Medicine, Mid-German Heart Centre, University Hospital, Martin Luther University Halle-Wittenberg, Halle (Saale), Germany; ^6^Institute of General Practice and Family Medicine, Interdisciplinary Centre for Health Sciences, Medical Faculty of the Martin Luther University Halle-Wittenberg, Halle (Saale), Germany; ^7^Department of Internal Medicine II, Martin Luther University Halle-Wittenberg, Halle (Saale), Germany; ^8^Paediatric Haematology and Oncology, Martin Luther University Halle-Wittenberg, Halle (Saale), Germany; ^9^Institute for Medical Sociology, Martin Luther University Halle-Wittenberg, Halle (Saale), Germany; ^10^Department of Internal Medicine I, Martin Luther University Halle-Wittenberg, Halle (Saale), Germany; ^11^Julius-Bernstein-Institute of Physiology, Medical Faculty of the Martin Luther University Halle-Wittenberg, Halle (Saale), Germany

**Keywords:** anxiety, armed conflicts, cohort studies, depression, psychological distress

## Abstract

**Introduction:**

The Russian invasion of Ukraine and the resulting consequences are in the center of political discussions, media, and likely individual thinking of the population in Germany. Yet, the impact of this prolonged exposure on mental health is not known hitherto.

**Methods:**

Using the population based cohort study DigiHero from three federal states (Saxony-Anhalt, Saxony, and Bavaria), we assessed anxiety levels (GAD-7), depressive symptoms (PHQ-9), and distress (modified PDI) in the first weeks of war and 6 months later.

**Results:**

Of those 19,432, who responded in the first weeks of war, 13,934 (71.1%) responded also 6 months later. While anxiety and emotional distress decreased during the 6 months, their average scores were still elevated, and a substantial fraction of respondents displayed clinically relevant sequelae. Persons from low-income households were especially affected, specifically by fears related to the personal financial situation. Those who reacted with a particularly strong fear in the beginning of war were more likely to have persistent clinically relevant symptoms of depression and anxiety also 6 months later.

**Discussion:**

The Russian invasion of Ukraine is accompanied by continuing impairment of mental health in the German population. Fears surrounding the personal financial situation are a strong determinant.

## 1. Introduction

Mass traumatic events, such as wars, have extensive mental health implications especially on those immediately affected (1). A war has been shown to lead to higher prevalence of anxiety, mood disorders and posttraumatic stress disorder (PTSD) in the general population of the affected country during and for years post-war (2, 3). The ongoing war in Ukraine has already been shown to have detrimental impacts on the mental health of Ukrainian military and civilian combatants (4) and the Ukrainian general population (5) with nearly half of the participants in both studies displaying clinically relevant symptoms of depression and anxiety. Persons who are internally displaced due to the ongoing conflict between Russia and Ukraine are at an especially high risk for mental health sequelae (6, 7).

Additionally, evidence suggests an impact of mass traumatic events on the mental health of individuals and populations not directly involved. However, published studies were mostly focused on short-term events such as mass shootings and terror attacks. Furthermore, studies suggested that exposure through traditional and social media leads to increased symptoms of distress (8, 9).

Due to the prolonged duration, compared to punctual events, the consequences of war can be more severe and extensive and aggravate over time. Beyond immediate conflict, the trauma can include flight experiences. Moreover, economic changes, such as inflation, can result, with global impact. These aspects might be pathways through which the mental health of populations not immediately involved in the war might suffer (10, 11).

In a previous study, we found that the invasion of Ukraine by Russia had a strong impact on the mental health of the general population in Germany in the first days and weeks (12). The effect of the early stages of the war on mental health has also been shown in other countries not directly involved in the war. Taiwanese and Polish participants in a study by Chudzicka-Czupała et al. showed high levels of distress caused by media war scenes (5). Another study found high proportions of moderate to severe levels of anxiety and depression among college students in Czech Republic in the month after the invasion and symptoms were positively correlated to feeling concerned about the war (13). Fear of the war was also found to be negatively correlated with Quality of Life in the Romanian general population (14). The war also seems to be correlated with a worsening of symptoms of people already dealing with mental health issues in Denmark (15). The effects of the prolonged exposure to the war have not yet been studied.

Earlier work suggests the possibility of habituation to traumatic stress, leading to a decrease in negative emotional reactions (16, 17). This habituation could reduce the burden on mental health but also decrease the engagement. Specifically, charities have reported a decrease of donations related to war in the German population (18). Still, it is not known how fast the decrease of emotional burden occurs, to what extent it happens, and which variables are associated with specific patterns.

Therefore, the aim of this study was to assess mental health in the German population 6 months after the Russian invasion in Ukraine in comparison to the early weeks of the war.

## 2. Methods

We use data from the prospective cohort for digital health research in Germany (DigiHero, DRKS Registration-ID: DRKS00025600). The cohort was initiated in January 2021 in Halle (Saale) and since then expanded to 10 German federal states, with a total sample currently approaching 70,000 households.^1^ In the randomly selected districts, samples are derived from resident registries, and we invite persons aged 18 to 85 years from *ca.* 1/3 households. In cities above 100,000 inhabitants, we restrict the invited sample to 30,000. The response is regionally differing between 3 and 5%. The registration for the study and the subsequent questionnaires are offered online. Immediately after registration, participants receive a baseline questionnaire. The cohort employs three to four questionnaires or other data collection events per year and will become part of the newly established German Center for Mental Health.

The baseline assessment contains questions about age, sex, if participants were born within the current boarders of Germany, relationship status, net household income, and household composition (number, age and relation of all household members). Further, we ask for school, higher and vocational training and categorize education by the ISCED criteria from 1997 (19). Information on the state and area of residence is derived from recruitment information.

In the beginning of March 2022, 1 week after the Russian invasion of Ukraine the by then recruited 27,509 cohort participants from Saxony-Anhalt, Saxony, and Bavaria received a questionnaire on mental health and fears related to the impact of the war (T1). Six months later, from 5th of September to the 4th of October, the 19,432 participants who participated in the first survey received a follow-up questionnaire (T2), assessing the same aspects.

The T1 and T2 questionnaire measured anxiety and depressive symptoms with the respective modules of the Patient Health Questionnaire. The anxiety module (GAD-7) consists of seven items (e.g., “Feeling nervous, anxious or on edge,” “Not being able to stop or control worrying”) measuring symptom severity over the last 2 weeks on a scale from 0 (not at all) to 3 (nearly every day), leading to a score ranging from 0 to 21, with higher scores indicating higher symptom severity. The internal consistency of the instrument is excellent with a Cronbach alpha of 0.92 (20). The depression module (PHQ-9) uses the same scale and time reference for nine items (e.g., “Little interest or pleasure doing things,” “Feeling down, depressed, or hopeless”) leading to a score from 0 to 27. The PHQ-9 shows good internal consistency (Cronbach alpha = 0.89) (21). For both scales, a cut-off indicating clinically relevant symptoms has been established at a score of ≥10 (21, 22). The questionnaires are widely used in psychiatric and epidemiological studies.

Emotional and physical distress was measured with an adapted version of the Peritraumatic Distress Inventory (PDI) (23). The adaption is described elsewhere (12). In brief, the instrument has five items for emotional (e.g., “I feel helpless,” “I feel frustrated and angry about not being able to do more”) and four items for physical distress (e.g., “I have trouble concentrating,” “I have physical reactions like sweating, shaking, and my heart pounding”) each measured on 5-point Likert scale from not at all (0) to extremely true (4) scale. The Cronbach alpha for the emotional distress subscale is 0.85 and 0.79 for the physical distress subscale. The scores for emotional and physical distress are calculated by the mean across the items.

To measure the fears related to the impact of war, we modified an instrument assessing fear of different life events used in the German National Cohort, adding the fear of consequences of war and using a scaled response instead of dichotomous. Fear is measured on a 4-point scale from 0 (not at all) to 3 (very strong). In the follow-up questionnaire (T2) at 6 months, we added a question specifying the consequences of war participants are afraid of, including worsening of individual financial situation, expansion of military conflict to other countries, economic crisis, and the exacerbation of consequences of the climate crisis. This follow up question was asked only among participants who indicated any fears of war consequences.

We report the proportion of participants with clinically relevant symptoms of depression and anxiety at both time points and proportion of different aspects of fears. In addition, we studied how anxiety, depression, and distress were associated with age (stratified by sex) for both time points using generalized additive models as implemented in the R-package *mgcv* (24). To identify characteristics associated with fear of specific consequences of the war, multiple ordinal regression was used after proportional odds assumption was confirmed. Univariable and multivariable logistic regression models were employed to determine risk factors for persistent clinical relevant symptoms of depression and anxiety. Sociodemographic variables such as age, sex, migration background, education and region and characteristics of residence, which might affect the reaction to the events, as well as the fear of war at T1 were chosen as possible predictors for persistent clinically relevant symptoms.

The study adheres to the Helsinki Declaration and was approved by the Ethics Committee of the Martin Luther University Halle-Wittenberg. All participants of DigiHero provided informed consent for study participation.

## 3. Results

Out of the 19,432 respondents of the first survey, 13,934 (71.7%) answered the same questions 6 months later. Their characteristics are displayed in Table 1. Younger individuals, males, and participants with lower education were less likely to participate in the second survey, but the differences were minor (Supplementary Table S1). There were no substantial differences in mental health and fear at T1 between responders and non-responders to T2.

**Table 1:** Participants characteristics.

	*n* (%)
Overall	13,934 (100.0)
Age Groups^*^
0–29	1,198 (8.6)
30–39	2,214 (15.9)
40–49	2,355 (16.9)
50–59	3,451 (24.8)
60–69	3,103 (22.3)
70+	1,612 (11.6)
Sex*
Male	5,547 (39.8)
Female	8,374 (60.1)
Diverse	13 (0.1)
**Born in Germany**
Yes	13,539 (97.2)
No	370 (2.7)
Missing	25 (0.2)
**Education**
Low	489 (3.5)
Medium	4,075 (29.2)
High	8,786 (63.1)
Missing	584 (4.2)
**Net household income (in Euro)**
<1750	1815 (13.0)
1750–3,000	3,915 (28.1)
3,000–4,000	2,895 (20.8)
4,000–5,000	2,153 (15.5)
>5,000	2,134 (15.3)
Missing	1,022 (7.3)
**In partnership**
Yes	11,015 (79.1)
No	2,717 (19.5)
Missing	202 (1.4)
**Child < 18 living in household**
Yes	3,326 (23.9)
No	10,529 (75.6)
Missing	69 (0.5)
**State**
Saxony-Anhalt	9,693 (69.6)
Saxony-Anhalt	2049 (14.7)
Bavaria	1998 (14.3)
Others	168 (1.2)
Missing	26 (0.2)
**City**
Yes	6,146 (44.1)
No	7,633 (54.8)
Missing	155 (1.1)

*obligatory answers.

**Figure 1:**
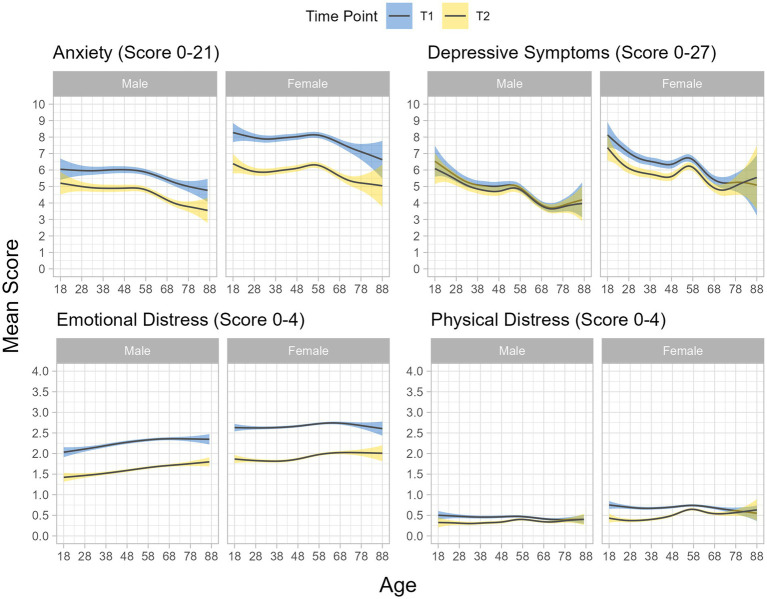
Average value of mental health indicators by age and sex* at the start of the war (T1) and 6 month later (T2). Figures are obtained by ggplot, applying the gam function with splines in mgcv library. *due to group size, analysis by age was only carried out for male and female sex.

While there was a decrease of anxiety and emotional distress from T1 to T2, a substantial level of mental health impairment persisted (Figure 1). The proportion of participants reporting clinically relevant symptoms of anxiety decreased by 11.3 percentage points from the measurement in early weeks of war (27.3%) to 6 months later (16%). The decrease in percentage of participants reporting clinically relevant symptoms of depression was not as pronounced (from 19.5% in the early weeks to 16.4% 6 months later).

The persistent clinically relevant symptoms of anxiety and depression were more common in women, persons with migration background, singles, for those living in households with children, and those with low income (Table 2). Fear of war at T1 was the strongest predictor for persistent clinically relevant symptoms of anxiety and depression.

**Table 2:** Multi-and univariable logistic regression models for predictors of persistent anxiety and depression.

	Anxiety		Depression	
	OR [95% Confidence Interval]			
	Univariable	Multivariable	Univariable	Multivariable
**Age** (in years)*
>50	1.009 [1.002; 1.016]	1.015 [1.006; 1.024]	0.999 [0.993; 1.005]	1.006 [0.999; 1.015]
<50	0.941 [0.925; 0.957]	0.922 [0.903; 0.941]	0.934 [0.913; 0.955]	0.912 [0.891; 0934]
**Sex**
Male	Ref.			
Female	1.93 [1.71; 2.19]	1.37 [1.19; 1.58]	1.72 [1.52; 1.95]	1.27 [1.10; 1.46]
Diverse	11.92 [3.82; 36.07]	4.83 [1.03; 22.68]	11.52 [3.69; 34.86]	3.07 [0.68; 13.74]
**Born in Germany**
Yes	Ref.			
No	1.19 [0.85; 1.63]	1.65 [1.15; 2.36]	1.25 [0.89; 1.71]	1.70 [1.18; 2.46]
**Education**
High	Ref.			
Medium	1.35 [1.20; 1.52]	1.08 [0.94; 1.24]	1.72 [1.52; 1.94]	1.29 [1.12; 1.48]
Low	1.44 [1.08; 1.89]	1.16 [0.80; 1.67]	2.23 [1.71; 2.87]	1.20 [0.86; 1.68]
**Net household income (in Euro)**
<1750	Ref.			
1750–3,000	0.65 [0.55; 0.77]	0.66 [0.55; 0.80]	0.53 [0.45; 0.62]	0.59 [0.50; 0.71]
3,000–4,000	0.59 [0.50; 0.71]	0.59 [0.47; 0.72]	0.45 [0.37; 0.53]	0.51 [0.41; 0.62]
4,000–5,000	0.49 [0.40; 0.60]	0.46 [0.36; 0.58]	0.36 [0.29; 0.44]	0.39 [0.31; 0.50]
>5,000	0.35 [0.28; 0.43]	0.34 [0.26; 0.44]	0.27 [0.21; 0.33]	0.30 [0.23; 0.39]
**In partnership**
Yes	Ref.			
No	1.38 [1.21; 1.57]	1.21 [1.03; 1.43]	1.83 [1.62; 2.08]	1.38 [1.18; 1.62]
**Child < 18 living in household**
No	Ref.			
Yes	1.12 [0.99; 1.27]	1.17 [0.99; 1.38]	0.94 [0.82; 1.07]	0.92 [0.78; 1.09]
**State**
Saxony-Anhalt	Ref.			
Saxony	0.96 [0.82; 1.12]	0.97 [0.81; 1.16]	0.88 [0.74; 1.03]	0.87 [0.72; 1.05]
Bavaria	0.78 [0.66; 0.93]	0.89 [0.73; 1.09]	0.92 [0.78; 1.08]	1.03 [0.84; 1.25]
Others	0.43 [0.19; 0.82]	0.84 [0.24; 2.85]	0.70 [0.37; 1.22]	0.82 [0.24; 2.81]
**City**
Yes	Ref.			
No	1.08 [0.97; 1.21]	1.05 [0.92; 1.20]	1.05 [0.94; 1.18]	1.11 [0.96; 1.27]
**Fear of war consequences at T1** [0–4]	2.43 [2.25; 2.64]	2.36 [2.16; 2.58]	1.76 [1.63; 1.89]	1.77 [1.63; 1.93]

*Segmented analysis with break-point = 50.

**Figure 2:**
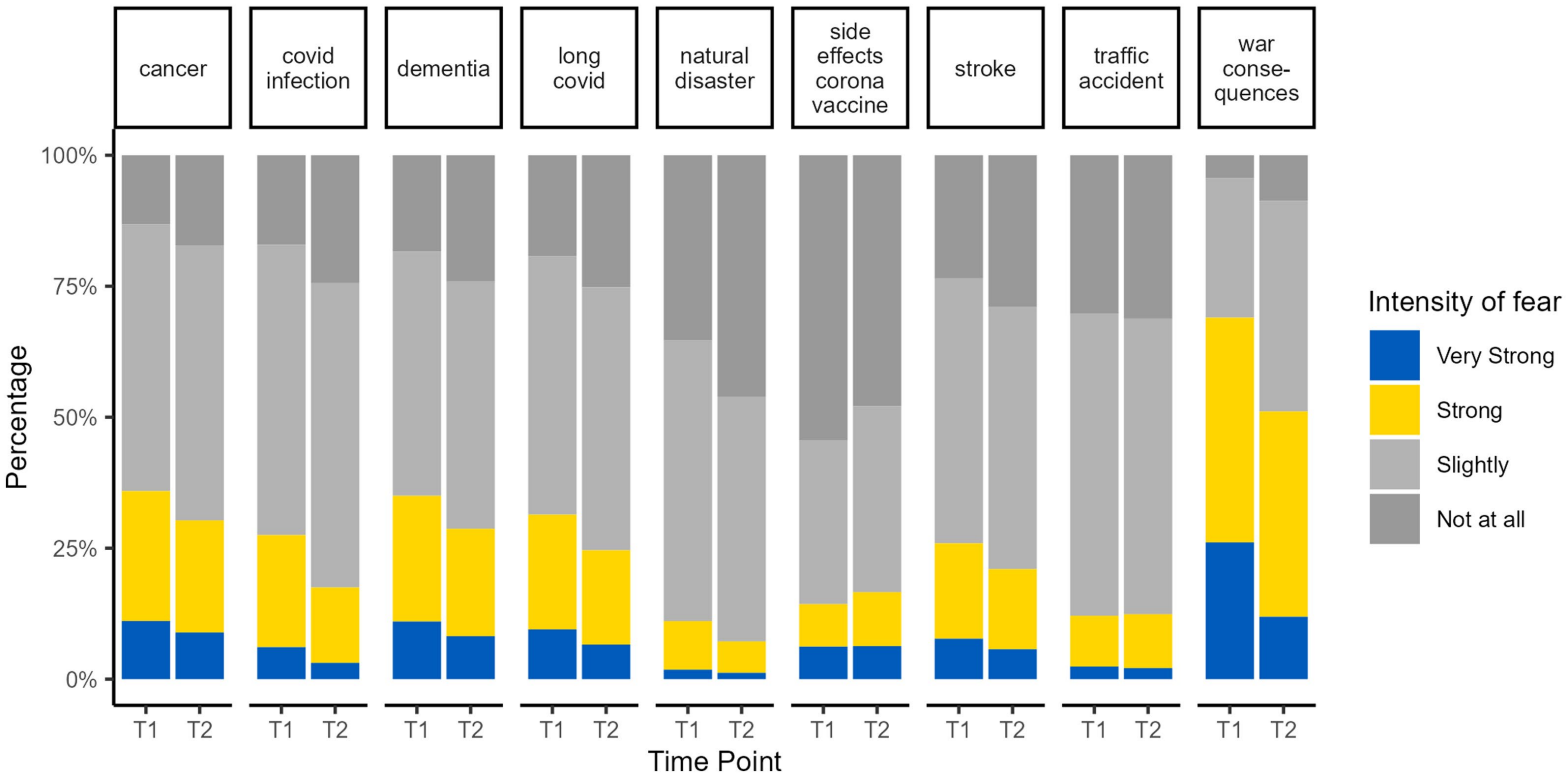
Fears of health related and other events at the start of the war (T1) and 6 month later (T2).

With respect to fears, there were minor reductions for most investigated diseases or events (Figure 2). In contrast, there was a substantial reduction for fears of war consequences. Nevertheless, the fear of war consequences was much higher than any other fear also 6 months after the beginning of war, and around half of the participants reported very strong or strong fear.

**Figure 3:**
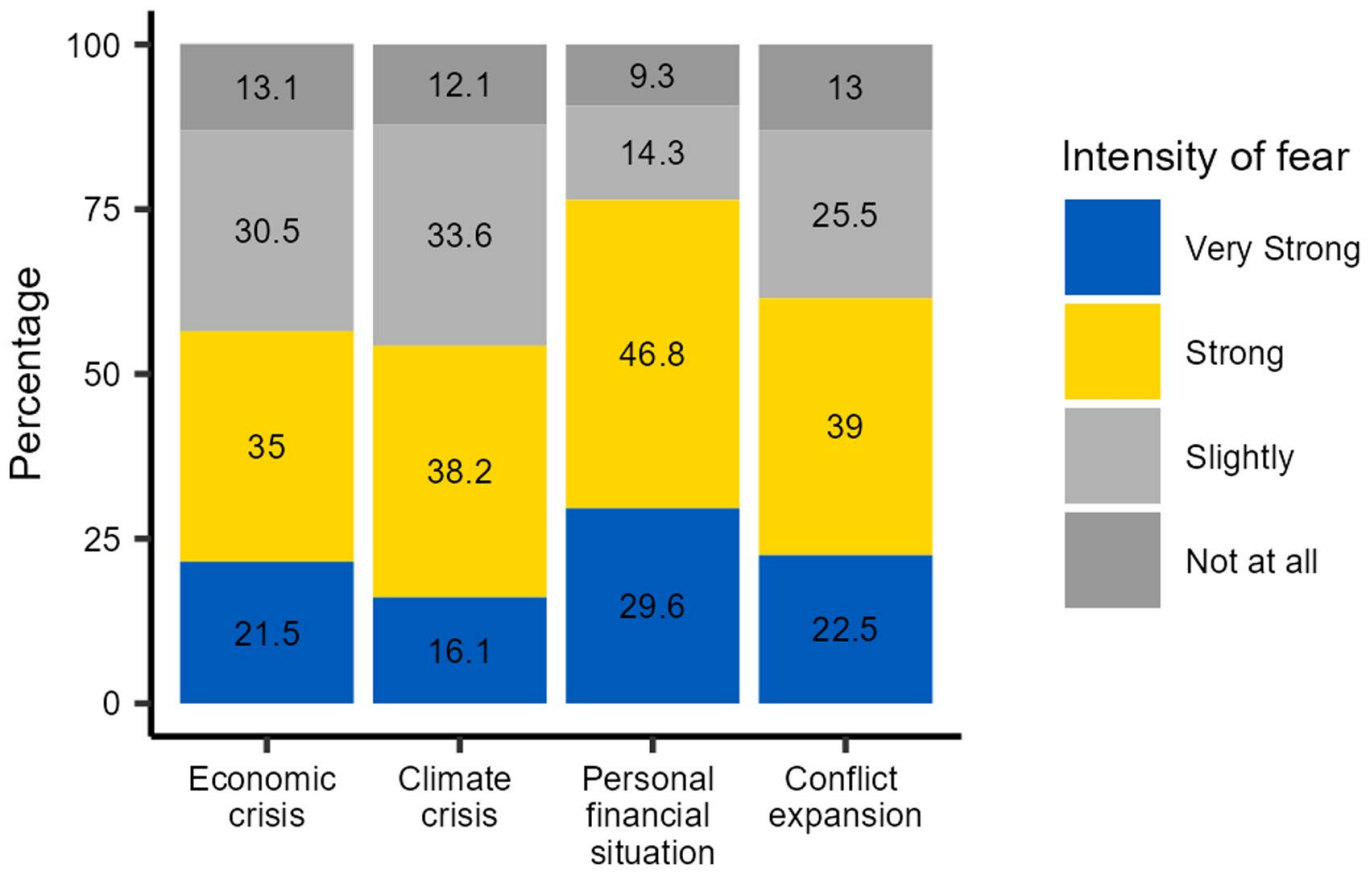
Specific fears related to war 6 month after the start of the war (T2).

At the T2, the dimensions of the fear of war consequences were asked in more detail (Figure 3). Fears of economic crisis, climate crisis and personal financial situation were all more pronounced than the fear of military expansion of the war. The most common fear was of economic crisis, with one third of the respondents reporting a very strong fear.

All fears related to war consequences were more pronounced in older people, except fears of worsening personal financial situations, which displayed no association with age (Table 3). Fears of conflict expansion were particularly pronounced among women. Consequences for personal financial situations displayed the strongest gradient across the income groups, with lesser effects in affluent households. Participants with children under 18 years were particularly affected by fears regarding personal financial situation in the context of war. Participants from Bavaria displayed substantially less fears in all assessed dimensions compared to Saxony-Anhalt and Saxony. Participants from rural regions were affected more strongly by all fears of consequences of war apart from those related to climate change. Education displayed no clear gradient for the fears.

**Table 3:** Multivariable ordinal regression for characteristics associated with specific aspects of fears.

	Economic crisis	Climate crisis	Financial situation	Conflict expansion
	OR [95%-CI]			
**Age** [per 10 years]	1.16 [1.13; 1.20]	1.15 [1.12; 1.18]	1.02 [1.00; 1.05]	1.25 [1.21; 1.28]
**Sex**
Male	Ref.			
Female	1.66 [1.55; 1.78]	1.82 [1.70; 1.95]	1.54 [1.44; 1.64]	2.42 [2.26; 2.60]
Diverse	3.89 [1.06; 14.76]	2.50 [0.75; 8.37]	3.9 [1.11; 14.45]	6.85 [1.90; 28.84]
**Born in Germany**
Yes	Ref.			
No	0.96 [0.78; 1.18]	1.07 [0.87; 1.32]	0.87 [0.71; 1.07]	0.99 [0.81; 1.23]
**Education**
High	Ref.			
Medium	1.17 [1.08; 1.26]	0.90 [0.84; 0.97]	1.36 [1.26; 1.47]	1.04 [0.96; 1.12]
Low	0.87 [0.71; 1.07]	1.02 [0.83; 1.26]	0.81 [0.66; 1.00]	0.84 [0.68; 1.03]
**Net household income**
<1750	Ref.			
1750–3,000	1.02 [0.91; 1.15]	0.93 [0.83; 1.04]	0.74 [0.66; 0.84]	0.98 [0.88; 1.10]
3,000–4,000	0.95 [0.84; 1.08]	0.86 [0.76; 0.97]	0.64 [0.57; 0.73]	0.88 [0.77; 0.99]
4,000–5,000	0.98 [0.86; 1.13]	0.85 [0.74; 0.97]	0.52 [0.46; 0.60]	0.89 [0.78; 1.01]
>5,000	0.84 [0.73; 0.96]	0.79 [0.69; 0.90]	0.35 [0.31; 0.40]	0.75 [0.65; 0.86]
**In partnership**
Yes	Ref.			
No	0.83 [0.75; 0.91]	0.76 [0.70; 0.84]	0.73 [0.67; 0.80]	0.80 [0.73; 0.88]
**Child < 18 living in household**
No	Ref.			
Yes	1.11 [1.01; 1.21]	0.92 [0.84; 1.00]	1.32 [1.21; 1.44]	0.97 [0.89; 1.05]
**State**
Saxony-Anhalt	Ref.			
Saxony	0.84 [0.76; 0.92]	0.79 [0.72; 0.87]	0.88 [0.80; 0.97]	0.91 [0.82; 1.00]
Bavaria	0.63 [0.57; 0.69]	0.86 [0.78; 0.95]	0.56 [0.51; 0.62]	0.68 [0.62; 0.76]
Others	0.72 [0.38; 1.39]	1.09 [0.57; 2.08]	0.72 [0.38; 1.36]	0.60 [0.32; 1.12]
**City**
Yes	Ref.			
No	1.35 [1.26; 1.45]	1.00 [0.93; 1.07]	1.45 [1.35; 1.55]	1.21 [1.13; 1.29]

## 4. Discussion

While the impairment of mental health was less pronounced after 6 months than immediately after the start of the war in Ukraine, elevated symptoms of anxiety and depression remained common. Most recent data on the proportion of people having clinically significant symptoms of anxiety and depression for the German population comes from the German National Cohort (NAKO), which has a sample with similar characteristics to our study sample (25). About 4.3 and 4.7% of NAKO participants displayed clinically relevant scores of anxiety before and at the beginning of the Covid-19 pandemic, respectively, and 6.4 and 8.8% of depression (26). The presented data show two to three times higher percentages of clinically relevant symptoms of anxiety and depression 6 months after the beginning of the war. Therefore, even if there is some indication of a less pronounced reaction than in the beginning of war, the impairment of mental health is still substantial.

Fear of war consequences was still the most prominent fear 6 months after the start of war in comparison to a range of other events or health related outcomes. In the beginning of the war, we did not distinguish between the various components, but 6 months later the economic fears were the leading component. Economic crises have been found to have a strong impact on population mental health, especially regarding depressive symptoms and anxiety, affecting groups less affluent more than others (10, 11). Therefore, the impact of war on mental health through triggering an economic crisis is plausible. Still, 6 months after the beginning of the war, a substantial fraction of the population feared the extension of the military conflict. This indicates the difficulty and complexity of the situation – some of the fears became more estimable as for example the increased living costs, while others remain potential and undefined.

We also found certain groups being more affected by continuing symptoms of anxiety, depression, distress, and by fear of the consequences of war. We already reported that general fear of war increased with age (12). The same pattern was observed for the specific fears of economic crisis, climate crisis, and conflict expansion. This is in contrast to the observations made during the Covid-19 pandemic, where higher age was a protective factor for mental health impairment (27). Women had higher risk of persistent depressive and anxiety symptoms and higher levels of fear of all war consequences. This is in line with the literature before and during the Covid-19 pandemic, where women always displayed higher symptom burden for anxiety, depression, and distress (28–30).

The fears of war consequences were higher in regions with Eastern bloc history, where even 30 years later past memories can be relevant. The higher fear of economic crisis in the Eastern States Saxony-Anhalt and Saxony, compared to Bavaria in West Germany, could further stem from the worse economic situation in Eastern Germany, with lower income (31) and higher unemployment (32).

Participants with lower household income had greater fear of worsening of their individual financial situation than those more affluent. In contrast, this correlation was not found for the fear of an economic crisis, even though this might especially affect low-income households (10). Apparently, general concerns related to an economic crisis might go beyond the situation involving the own precarious situation. Living in a low-income household was also identified as a risk factor for continuing clinically relevant symptoms of anxiety and depression. Hence, this group seems especially at risk of mental health impairment from the consequences of war.

Another vulnerable group were persons with migration background. We have a low proportion of persons with migration background in our sample, partly due to the recruitment regions and a high proportion of participants from rural regions. Thus, our sample is surely not representative of migrants living in Germany. Refugees from Ukraine and other countries are probably not represented in our study, however, this groups is expected to have an even higher burden of mental health impairment due to flight experiences and (33, 34) and at risk for re-traumatization by the continuing war (35).

### 4.1. Strength and limitations

The sample of DigiHero is population-based with respect to sampling, but in the composition not representative of the German population. Given the higher proportion of the better-educated being more likely to participate in health related studies, it is important that no education gradient was present for the studied questions. The sociodemographic characteristics of the DigiHero sample are similar to the participants of the NAKO cohort (25). In the NAKO study, participants received three postal invitations or were contacted by phone with a resulting response of *ca.* 20%. In an additional effort, potential respondents were even visited at home. In a previous analysis, we demonstrated that those recruited with the extensive efforts of home visits did not differ from those recruited before (36). In the DigiHero study, only a single invitation was sent, accepting a lower response at higher efficiency. With respect to online participation only, we demonstrated in an earlier study that across a large sample of health related questions responses provided in the online only arm did not differ from responses provided in a sample, in which paper-based participation was possible (37). Our sample is likely representative of the fraction of the population participating in epidemiologic studies. Beyond this group, achieving representativeness is and will be increasingly difficult (38). Still, Participants of DigiHero were recruited independently of the here studied question and among them the response to the first questionnaire was high. Similarly, the fraction responding to the second questionnaire was also high and participation was not conditional on higher values of anxiety or depressive symptoms in the first questionnaire. Furthermore, the invitation to the first questionnaire spoke only of a “current topic” and did not mention the war. Overall, we cannot exactly estimate the level of concern for the entire German population, but our data inform about a substantial public health problem in this context.

The big sample, with coverage of rural and urban regions from three federal states in Germany are strengths of the study. The timely collection of comparison data at the beginning of the war was an advantage for the assessment of the effect of war after 6 months of exposure. This comparison was possible with a high response proportion for online surveys (39). Thus, we are confident of the relevance of our results and their contribution to mental health monitoring during prolonged mass traumatic events. The continuous assessment of mental health indicators is important and should include other factors, such as the media exposure.

### 4.2. Conclusion

While an impairment of mental health related to the Russian invasion of Ukraine decreased, the negative effects persisted at a high level 6 months after the beginning of the war. Fear of the consequences of war still dominated across a range of potential fears. The concerns were likely more differentiated then in the beginning of war. Less affluent persons were more affected by fears regarding their personal financial situation related to the war than more affluent persons. The findings are relevant on a global level, as studies conducted immediately after the start of the war demonstrate a significant impact on mental health in countries beyond Ukraine and its neighboring countries (5, 15). Our study contributes to this body of knowledge by showing that the effects persist for up to 6 months after the initial shock of the war and may result in persistent mental health sequelae, particularly for already vulnerable groups.

## Data availability statement

The raw data supporting the conclusions of this article will be made available by the authors, without undue reservation.

## Ethics statement

The studies involving human participants were reviewed and approved by Ethik-Kommission der Medizinischen Fakultät der Martin-Luther-Universität Halle-Wittenberg (ethics vote: 2020-076). The patients/participants provided their written informed consent to participate in this study.

## Author contributions

JM, SD, BK, DC, CG, and RM developed the questionnaire, conducted the analyses, and drafted the manuscript. AB and OP were responsible for data curation. NO provided expert knowledge in the field of psychiatry. MB, DS, TF, MG, JH, IM, JR, MG, and RM developed the design of the DigiHero study. All authors provided comments on the manuscript and accepted the final version.

## Funding

There was no specific funding for the conducted survey. The DigiHero study is funded by internal resources of the Medical Faculty of the Martin Luther University Halle-Wittenberg and part of the recruitment was co-funded by the Ministry of Economy, Science and Digitalization of the Federal State of Saxony-Anhalt (Germany).

## Conflict of interest

The authors declare that the research was conducted in the absence of any commercial or financial relationships that could be construed as a potential conflict of interest.

## Publisher’s note

All claims expressed in this article are solely those of the authors and do not necessarily represent those of their affiliated organizations, or those of the publisher, the editors and the reviewers. Any product that may be evaluated in this article, or claim that may be made by its manufacturer, is not guaranteed or endorsed by the publisher.

## Footnotes

^1^www.medizin.uni-halle.de/digihero
