# Supplementary material for: Anxiety, depressive symptoms, and distress over the course of the war in Ukraine in three federal states in Germany
Source: Front Psychiatry. 2023 Apr 27;14:1167615. doi: 10.3389/fpsyt.2023.1167615 (PMC10172594; doi:10.3389/fpsyt.2023.1167615)
Supplement: Supplementary file 1 [file Table_1.DOCX]

Supplementary Material

Anxiety, depressive symptoms and distress over the course of the war in Ukraine in three federal states in Germany

Janka Massag, Sophie Diexer, Bianca Klee, Daniela Costa, Cornelia Gottschick, Anja Broda, Oliver Purschke, Nils Opel, Mascha Binder, Daniel Sedding, Thomas Frese, Matthias Girndt, Jessica Hoell, Irene Moor, Jonas Rosendahl, Michael Gekle, Rafael Mikolajczyk^*^

*** Correspondence:** Rafael Mikolajczyk: Rafael.mikolajczyk@uk-halle.de

# Supplementary Table

**Table S1: Characteristics of participants who answered the follow-up (T2) questionnaire and participants that only answered the first questionnaire (T1)**

|  | **T2 Responder** | **T2 Non-Respnder** |
| --- | --- | --- |
| **n** | 13934 | 4613 |
| **Age group** |  |  |
| 0-29 | 1198 (8.6) | 786 (17.0) |
| 30-39 | 2214 (15.9) | 908 (19.7) |
| 40-49 | 2355 (16.9) | 769 (16.7) |
| 50-59 | 3451 (24.8) | 887 (19.2) |
| 60-69 | 3104 (22.3) | 731 (15.8) |
| 70+ | 1612 (11.6) | 532 (11.5) |
| **Sex** |  |  |
| Male | 5547 (39.8) | 2081 (45.1) |
| Female | 8374 (60.1) | 2526 (54.8) |
| Diverse | 13 (0.1) | 6 (0.1) |
| **Born in Germany** |  |  |
| Yes | 13539 (97.2) | 4450 (96.5) |
| No | 370 (2.7) | 151 (3.3) |
| Missing | 25 (0.2) | 12 (0.3) |
| **Education** |  |  |
| Low | 489 (3.5) | 326 (7.1) |
| Medium | 4075 (29.2) | 1437 (31.2) |
| High | 8786 (63.1) | 2644 (57.3) |
| Missing | 584 (4.2) | 206 (4.5) |
| **Net household income** |  |  |
| <1750 | 1815 (13.0) | 638 (13.8) |
| 1750 - 3000 | 3915 (28.1) | 1279 (27.7) |
| 3000 - 4000 | 2895 (20.8) | 919 (19.9) |
| 4000 - 5000 | 2153 (15.5) | 624 (13.5) |
| >5000 | 2134 (15.3) | 739 (16.0) |
| Missing | 1022 (7.3) | 414 (9.0) |
| **In partnership** |  |  |
| Yes | 11015 (79.1) | 3657 (79.3) |
| No | 2717 (19.5) | 864 (18.7) |
| Missing | 202 (1.4) | 92 (2.0) |
| **Child <18 in household** |  |  |
| Yes | 3326 (23.9) | 1221 (26.5) |
| No | 10539 (75.6) | 3362 (72.9) |
| Missing | 69 (0.5) | 30 (0.7) |
| **State** |  |  |
| Saxony-Anhalt | 9693 (69.6) | 3003 (65.1) |
| Saxony | 2049 (14.7) | 733 (15.9) |
| Bavaria | 1998 (14.3) | 815 (17.7) |
| Others | 168 (1.2) | 49 (1.1) |
| Missing | 26 (0.2) | 13 (0.3) |
| **City** |  |  |
| Yes | 6146 (44.1) | 1914 (41.5) |
| No | 7633 (54.8) | 2646 (57.4) |
| Missing | 155 (1.1) | 53 (1.1) |
| **Mean GAD-7 Score at T1** | 7.07 (4.95) | 7.02 (4.89) |
| **Mean PHQ-9 Score at T1** | 5.73 (4.86) | 6.04 (5.07) |
| **Mean Emotional Distress Score at T1** | 2.52 (0.91) | 2.44 (0.94) |
| **Mean Physical Distress Score at T1** | 0.60 (0.76) | 0.61 (0.77) |
| **Fear of war consequences at T1** |  |  |
| Not at all | 612 (4.4) | 258 (5.6) |
| Slightly | 3699 (26.5) | 1287 (27.9) |
| Strong | 5968 (42.8) | 1930 (41.8) |
| Very Strong | 3627 (26.0) | 1106 (24.0) |
| Missing | 28 (0.2) | 32 (0.7) |
